# Render-Rank-Refine: Accurate 6D Indoor Localization via Circular Rendering

**DOI:** 10.3390/jimaging12010010

**Published:** 2025-12-25

**Authors:** Haya Monawwar, Guoliang Fan

**Affiliations:** School of Electrical and Computer Engineering, Oklahoma State University, Stillwater, OK 74078, USA; haya.monawwar@okstate.edu

**Keywords:** indoor localization, layout ambiguity, pose estimation, rotation-invariant descriptors, semantic models

## Abstract

Accurate six-degree-of-freedom (6-DoF) camera pose estimation is essential for augmented reality, robotics navigation, and indoor mapping. Existing pipelines often depend on detailed floorplans, strict Manhattan-world priors, and dense structural annotations, which lead to failures in ambiguous room layouts where multiple rooms appear in a query image and their boundaries may overlap or be partially occluded. We present *Render-Rank-Refine*, a two-stage framework operating on coarse semantic meshes without requiring textured models or per-scene fine-tuning. First, panoramas rendered from the mesh enable global retrieval of coarse pose hypotheses. Then, perspective views from the top-*k* candidates are compared to the query via rotation-invariant circular descriptors, which re-ranks the matches before final translation and rotation refinement. Our method increases camera localization accuracy compared to the state-of-the-art SPVLoc baseline by reducing the translation error by 40.4% and the rotation error by 29.7% in ambiguous layouts, as evaluated on the Zillow Indoor Dataset. In terms of inference throughput, our method achieves 25.8–26.4 QPS, (Queries Per Second) which is significantly faster than other recent comparable methods, while maintaining accuracy comparable to or better than the SPVLoc baseline. These results demonstrate robust, near-real-time indoor localization that overcomes structural ambiguities and heavy geometric assumptions.

## 1. Introduction

Indoor camera localization, which is estimating a camera’s 6-degree-of-freedom (6-DoF) pose in complex indoor spaces, is a core challenge in computer vision with applications in navigation [1,2], augmented reality [3], and assistive technologies for people with disabilities [4]. Classical Structure-from-Motion and geometric methods [5,6] achieve high accuracy under controlled conditions but assume Manhattan world layouts or dense three-dimensional reconstructions. On the other hand, learning-based approaches such as PoseNet [7] offer greater flexibility but struggle in visually ambiguous scenarios. Moreover, traditional methods rely on dense scene-specific data including curated images, depth maps, or three-dimensional point clouds, limiting scalability. Lastly, although synthetic augmentation and simplified three-dimensional representations such as Structured3D [8] improve applicability, most methods focus on two-dimensional localization, which still leaves critical degrees of freedom unmodeled for 6-DoF poses.

Recent methods such as SPVLoc [9] and LASER [10] have advanced indoor pose estimation. SPVLoc does so by directly linking perspective queries to rendered semantic panoramas and achieves robust localization in unseen indoor layouts while LASER builds on this idea with a Monte Carlo inference pipeline and a geometrically organized latent space, rendering circular descriptors from floor-plan codebooks to deliver efficient and precise indoor localization. However, the assumption of largely unobstructed views, and correlation-based refinement is sensitive to the top-ranked panorama, so a single retrieval error can derail the estimate. Furthermore, heavy reliance on detailed and accurate floorplan annotations (such as window placements) and the 2D abstraction can limit performance under incomplete geometry or mismatched fields of view. While semantic retrieval methods provide scene-level robustness, they collapse under ambiguity. On the other hand, geometry-driven methods provide invariances to viewpoint, orientation, and appearance changes, but may still struggle without precise window-door-opening (WDO) annotations on the room layout. Furthermore, accurate floorplan registration is rarely feasible in practice, especially when on-the-fly pose estimation is needed in safety-critical settings such as caring for people with disabilities [4]. Hence, reliable camera localization is generally possible in environments with strong textures and distinctive room geometry.

**Figure 1:**
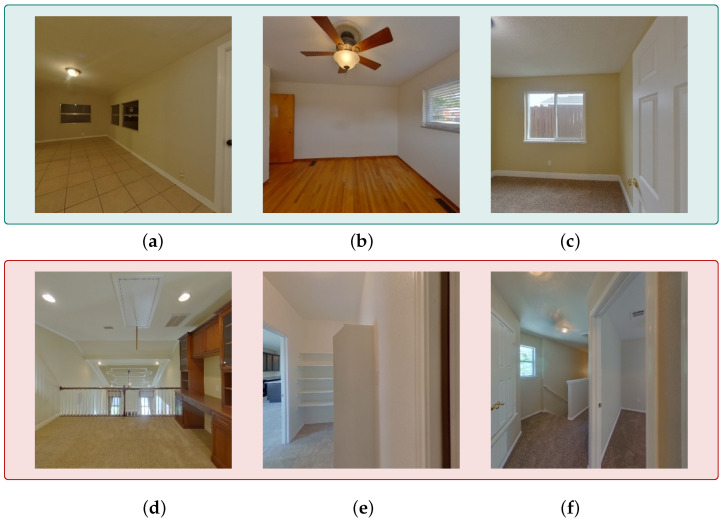
Examples from ZInD [12]. (**a**–**c**) represent visually clear layouts; (**d**–**f**) represent ambiguous or challenging cases.

In Figure 1, we illustrate how different image categories affect indoor localization. Figure 1a–c show relatively easy cases: (a) is an open tiled room with clear wall-floor boundaries and window geometry, providing strong geometric cues for camera localization; and (b) and (c) are bedrooms with distinctive elements such as a ceiling fan, and wood floor/carpet texture, all of which introduce unique orientation cues that simplify pose estimation. In contrast, Figure 1d,e represent challenging cases. They depict layout ambiguity, where multiple rooms appear within the same query image and their boundaries may overlap or be partially occluded. At the same time, rendering from the mesh model at that viewpoint often produces less well-defined room boundaries (e.g., indistinct ending points) in the rendered image, which introduces ambiguity in the extracted descriptors and creates challenges for accurate camera pose estimation. Hence, the extracted descriptors fail to map to a unique spatial location, and several candidate viewpoints in the model may produce indistinguishable renderings under the same mesh. This turns layout ambiguity into a persistent obstacle for reliable 6-DoF camera pose estimation.

**Figure 2:**
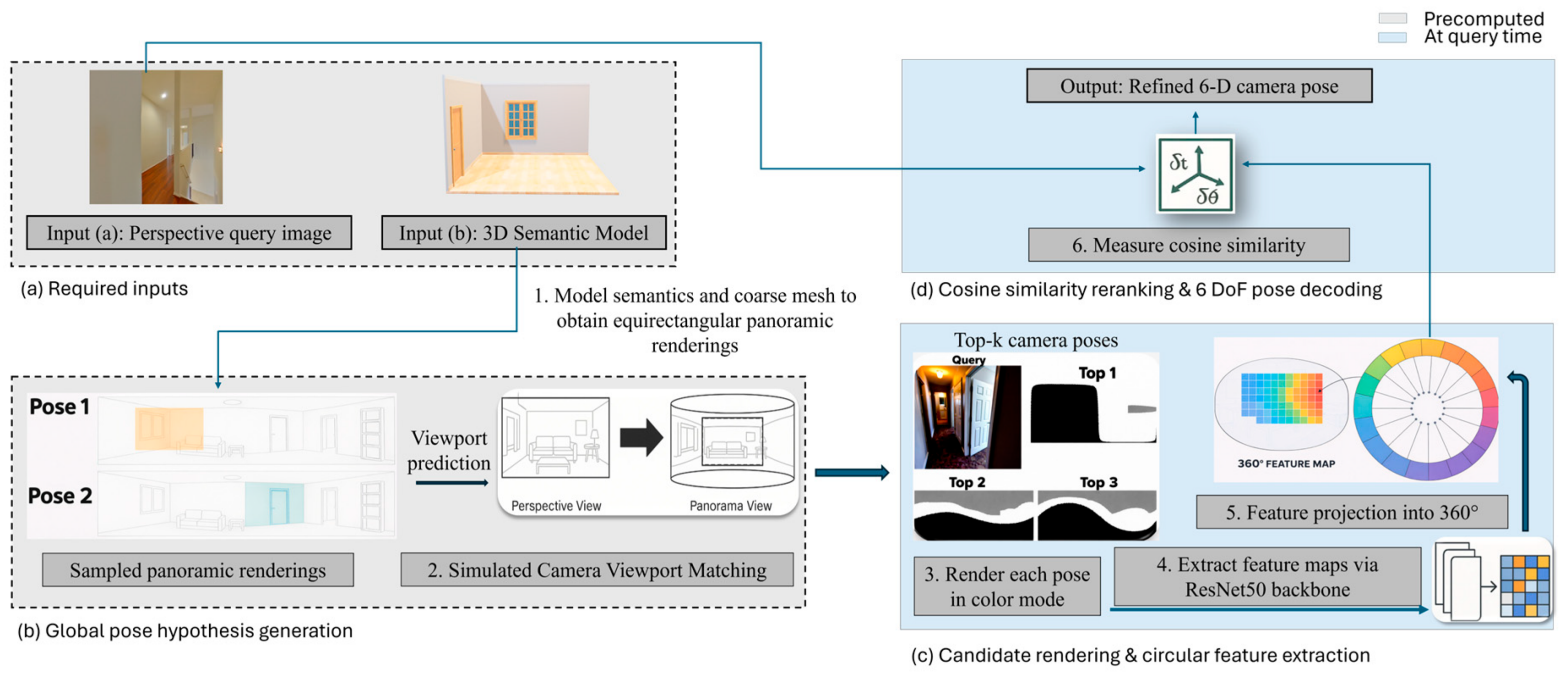
Overview of Render-Rank-Refine. (**a**) Panoramic views are pre-rendered from the 3D semantic model and compared with the perspective query image to generate candidate camera poses using viewport matching. (**b**) The top-ranked candidate poses are re-rendered in color, and feature maps are extracted using a ResNet-50 network. (**c**) These feature maps are then converted into circular 360-degree descriptors. (**d**) Cosine similarity is used to compare the query features with each descriptors from each pose, and the poses are ranked to obtain the final refined 6-DoF camera pose.

In this paper, we introduce Render-Rank-Refine, a 6-DoF camera localization framework that requires no scene-specific training or exhaustive annotations. It handles varying camera FoVs and mitigates errors from incorrect top-ranked candidates. The method integrates semantic and geometric representations to achieve superior accuracy in ambiguous indoor environments (see Figure 1). The proposed pipeline is illustrated in Figure 2. First, a perspective query and a 3D semantic model generate top-*K* candidate poses via viewport matching on rendered panoramas (from (a) to (b)). Then, each candidate is re-rendered, encoded into $360^{{}^{\circ}}$ circular descriptors using a ResNet50 backbone (step (c)) [11]., and ranked by cosine similarity (step (d)), yielding a refined 6-DoF pose.

Our contributions are fourfold: (1) formalization of layout ambiguity and its connection to pose failures, enabling a principled analysis of when and why camera localization may break down; (2) Bayesian refinement using mesh-segmentation retrieval as prior and rotation-invariant circular descriptors as likelihood; (3) group-theoretic analysis showing marginalization over SO(2)⊂SE(3) removes in-plane rotation while preserving global layout cues; and (4) comprehensive evaluation on the Zillow Indoor Dataset (ZInD) [12] with ambiguity-stratified analysis. To the best of our knowledge, this is the first work to formalize and address layout ambiguity in indoor pose estimation via principled Bayesian integration of complementary representations, and achieve a highly substantial pose estimation improvement in cases with high layout ambiguity via a computationally light-weighted pipeline. Our pipeline estimates an accurate 6-DoF camera pose with minimal query-time overhead of approximately 3.5 ms and maintains high performance in visually ambiguous environments.

## 2. Related Work

Robust 6-DoF indoor localization draws on four complementary directions: global retrieval, local features, view synthesis, and hybrid geometry-learning. Retrieval prunes candidates, local features capture spatial detail, rendering aligns real and synthetic views, and hybrid methods fuse learning with geometry. Studying these approaches exposes limitations in accurate camera pose estimation due to layout ambiguity, viewpoint variation, and retrieval errors, directly motivating our framework.

### 2.1. Image Retrieval-Based Localization

Global retrieval pipelines compress each image into a compact, learnable descriptor and leverage fast nearest-neighbor searches to shortlist pose candidates before any detailed matching. One such work is DenseVLAD [13], which aggregates hand-crafted local features into a single robust vector, boosting place-recognition recall across varied viewpoints. Patch-NetVLAD [14] builds on this by fusing multi-scale CNN features into one unified VLAD descriptor, markedly improving indoor retrieval accuracy. Lastly, Hierarchical Localization [15] divides the process into a coarse retrieval stage followed by fine-grained local-feature verification, reducing search complexity while preserving precision.

Despite their efficiency in pruning candidates, these methods lack the spatial granularity needed to recover precise 6-DoF poses in visually ambiguous or overlapping room scenarios, where similar global descriptors may correspond to distinct physical locations.

### 2.2. Learned Local Feature Matching

Classical local descriptors, such as SIFT [16], SURF [17], ORB [18], and BRISK [19], pioneered robust keypoint matching under challenging illumination and viewpoint variations. More recently, end-to-end frameworks like ASLFeat [20] have shown that jointly learning keypoint detection and description can yield superior repeatability and distinctiveness. However, accurate 6-DoF pose estimation remains dependent on geometric optimization, often using RANSAC for outlier rejection [21] and efficient PnP solvers [22]. These solvers require sufficient inlier correspondences, which can be difficult to obtain in low-texture or repetitive layout environments, especially in the presence of layout ambiguity.

### 2.3. Rendering and View Synthesis Methods

Synthesizing candidate views from proxy geometry helps bridge the domain gap between real RGB queries and CAD style models. SPVLoc renders semantic panoramas from coarse meshes for learned embedding matching [9], while LASER learns a latent rendering space from accurate floorplans [10]. LaLaLoc [23] and LaLaLoc++ [24] extend this with neural floorplan understanding and implicit 3D hallucination, reducing annotation needs and increasing throughput, though without explicitly modeling ambiguity. Scene reconstruction pipelines, such as SLAM++ [25], LSD-SLAM [26], ORB-SLAM/ORB-SLAM2 [27,28], ElasticFusion [29], and SemanticFusion [30] produce dense, semantically annotated maps for relocalization. More recent methods include Neural Radiance Fields (NeRF) for photorealistic novel view synthesis from sparse captures [31] and ScanComplete for volumetric completion of partial scans [32]. These approaches, while robust, typically depend on extensive scanning or precise annotations, limiting deployment in unmodeled or dynamic environments.

### 2.4. Hybrid Geometry-Learning Approaches

Differentiable pipelines integrate learned features with geometric modules for end-to-end pose estimation. PoseNet [7] pioneered single-image 6-DoF relocalization with a CNN, MapNet [33] added geometry-aware consistency, VidLoc [34] employed LSTMs for temporal smoothing, CNN-SLAM [35] incorporated learned monocular depth, DSAC [36] made RANSAC differentiable, and SVO [37] achieved fast sparse alignment.

Existing methods fall short due to (1) lack of explicit modeling of layout ambiguity; (2) sensitivity to in-plane rotation; and (3) refinement that assumes correctness of the top retrieval. Our Render-Rank-Refine framework addresses these gaps by uniting high-recall semantic retrieval with rotation-invariant circular descriptors where retrieval scores serve as priors and circular similarity as a likelihood, enabling a Bayesian update that systematically improves localization under ambiguity.

## 3. Methodology

**Figure 3:**
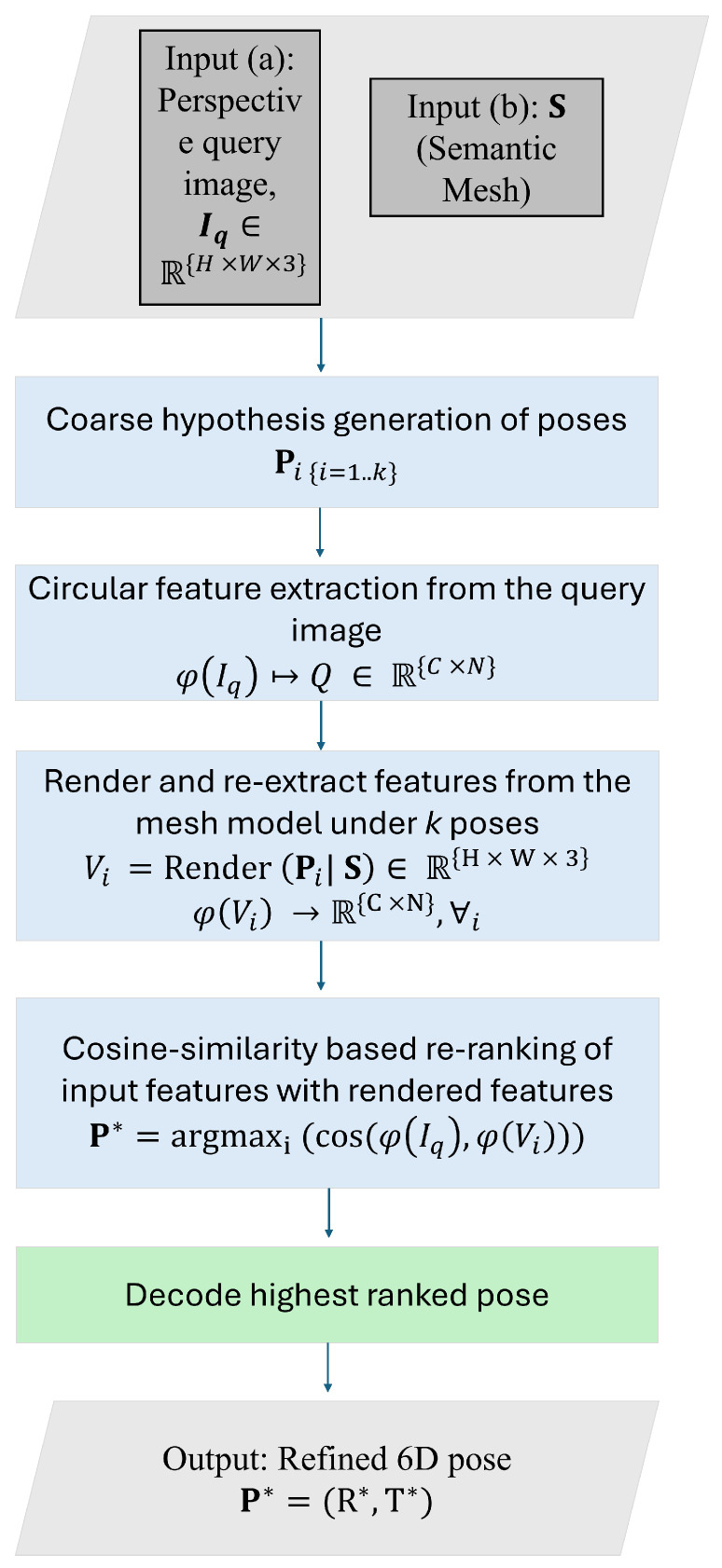
Overview of the proposed circular re-ranking pipeline. Given a query image and a semantic mesh, the pipeline generates coarse pose hypotheses, re-renders each candidate for descriptor-based re-ranking, extracts circular descriptors from the query image and renderings and compares their similarity, and finally decodes the highest-ranked hypothesis into a refined 6-DoF camera pose.

In this section, we present our framework for robust, efficient pose estimation in ambiguous indoor layouts. The pipeline consists of four main stages, two executed just once offline and two executed online in real time. The algorithm is illustrated in Figure 3. All modules are parallelized using multi-threaded computation.

Figure 3 follows the notation defined here. The query image is denoted by $I_{q}$, and the input semantic mesh by *S*. The image $I\in R^{H\times W\times 3}$ represents an RGB picture of height *H*, width *W*, and three color channels (RGB). The retrieval stage produces *k* coarse pose hypotheses $\left\{P_{i}\right\}$, where each pose $P_{i}=\left(R_{i},T_{i}\right)$ specifies a 6-DoF camera rotation and position. The circular descriptor of the query image is written as φ(I), and each candidate pose $P_{i}$ is re-rendered to produce a 2D view of the 3D mesh, *S*, $V_{i}=\mathrm{Render}\left(P_{i}|S\right)$. We extract descriptors $\varphi (V_{i})$ from these views and compare them to the query descriptors using cosine similarity. The highest-ranked candidate is selected as the final pose estimate, $P^{*}=\left(R^{*},T^{*}\right)$. This pipeline corresponds to stages (a)–(d) shown in Figure 2.

### 3.1. Problem Definition

Let $I_{q}$ denote a query RGB image captured at an unknown camera pose P∈SE(3), where SE(3) represents the group of all 3D rigid-body transformations (three translational and three rotational degrees of freedom). Given a known semantic 3D model of the environment S, the goal of indoor localization is to estimate the true pose P as accurately and efficiently as possible, while remaining robust to layout ambiguity.

Formally, this can be expressed as a maximum a posteriori (MAP) estimation problem:

(1) $$\hat{P}=\arg\max_{P\in SE(3)}p\left(P|I_{q},S\right),$$

where $p(P|I_{q},S)$ is the posterior probability of a candidate pose given the query image and the known scene. This formulation emphasizes that an ideal estimator should not only retrieve the most probable pose but should also do so in a way that accounts for ambiguities, is computationally tractable for large-scale environments, and generalizes to diverse indoor layouts. In our formulation, the retrieval stage provides an empirical prior p(P|S) based on semantic viewport correlations, while the circular descriptors supply an independent likelihood term $p(I_{q}\;|\;P,S)$ that measures view-consistency. We assume conditional independence between these two sources of evidence because they operate on different and complementary modalities: retrieval relies on dense semantic-geometric correlations in panoramic renderings, whereas the descriptor likelihood is driven by rotation-invariant global layout cues. This assumption makes the Bayesian update in Equation (1) coherent and allows the likelihood to correct retrieval bias by elevating low-ranked but geometrically consistent hypotheses.

### 3.2. Quantifying Layout Ambiguity

Indoor environments often contain regions that are visually and structurally similar, such as parallel corridors, identical utility rooms, or mirrored floorplans, making them challenging to disambiguate using image descriptors alone. To reason about this analytically, we define a pose ambiguity score for a given viewpoint *v* as

(2) $$A\left(v\right)=\frac{\langle p(v),p(u)\rangle }{∥p(v)∥\times ∥p(u)∥},$$

where p(·) denotes the descriptor representation of a view. Here, p(·) is the L2-normalized circular descriptor/feature of viewpoint *v*, constructed using the ResNet backbone, polar sampling, and ring-wise pooling described in Section 3. Each viewpoint corresponds to an image (query or rendered), from which a circular descriptor p(v) or p(u) is extracted for similarity comparison. This score measures the highest normalized similarity between *v* and any other distinct viewpoint *u* in the environment.

A high A(v) indicates that the view shares strong visual or structural resemblance with at least one other location, which increases the likelihood of retrieval errors and pose estimation failures. Empirical testing shows that failure rates increase significantly with the similarity ratio A(v), confirming its value as a predictor of localization difficulty.

### 3.3. Theoretical Motivation: A Bayesian View

Our approach treats pose estimation not as a single-step matching problem, but as a sequential process of hypothesis generation and hypothesis verification. This naturally lends itself to a two-stage Bayesian inference formulation, in which each stage contributes complementary information about the unknown camera pose.

Formally, we express the posterior distribution over the 6-DoF pose P∈SE(3), given a query image $I_{q}$ and a semantic 3D model S, as

(3) $$p\left(P|I_{q},S\right)\propto p\left(I_{q}|P,S\right)\times p\left(P|S\right).$$

Here, p(P∣S) reflects our prior belief about plausible poses before observing $I_{q}$, while $p(I_{q}|P,S)$ measures how well a hypothesized pose explains the observed image.

- Phase 1—Retrieval as the Prior
  We first use semantic mesh segmentation and viewport matching to rapidly narrow the search space from potentially thousands of poses to a small, high-recall set ${\left\{P_{i}\right\}}_{i=1}^{K}$. The retrieval scores $s_{i}$ assigned to these candidates are proportional to $p(P_{i}|I_{q},S)$ and thus act as an empirical prior. To construct the empirical prior p(P∣S), the retrieval correlation scores are passed through a softmax, which provides a monotonic and normalized mapping from raw similarity values to probabilities. Monotonicity ensures that if a candidate pose has a higher retrieval score $s_{i}$ than another $s_{j}$, then its prior probability $p(P_{i}|S)$ will also be higher, preserving the ranking produced by the retrieval stage. Normalization is necessary for the Bayesian update in Equation (5), where this probability-weighted prior is combined with the descriptor-based likelihood to form the final posterior.
- Phase 2—Descriptor Matching as the Likelihood
  We then introduce an independent source of evidence by computing rotation-invariant circular descriptors ϕ(·) for both the query and each candidate rendering $R(P_{i})$. The cosine similarity $c_{i}$ between $\phi (I_{q})$ and $\phi (R\left(P_{i}\right))$ measures how consistent the global layout is between the two views, regardless of in-plane rotation. We model this as a likelihood term:
  (4) $$p\left(P_{i}|I_{q},\phi \right)\propto \exp\left(c_{i}\right).$$
  The use of $\exp(c_{i})$ ensures that all likelihood weights are positive and that stronger matches are amplified in a smooth and monotonic manner [38]. This follows the standard practice in MAP estimation and log-linear or energy-based models [39], where compatibility scores are exponentiated to produce unnormalized likelihoods prior to the final posterior normalization. We keep this likelihood unnormalized to preserve independence across candidates. Applying a full softmax at this stage would force the candidates to compete and tie each weight to all others. Instead, normalization is performed only in the posterior (Equation (5)), after combining the retrieval prior and the descriptor-based likelihood.
  The final posterior probability for each candidate pose is then
  (5) $$p\left(P_{i}|I_{q},S,\phi \right)\propto p\left(P_{i}|I_{q},S\right)\times p\left(P_{i}|I_{q},\phi \right).$$
  This Bayesian update ensures that candidates supported by both the high-recall retrieval prior and the ambiguity-resolving descriptor likelihood rise to the top. Importantly, it allows correct poses that were initially ranked low due to retrieval bias to overtake incorrect top-1 candidates. Because the posterior is proportional to the product of the retrieval prior and the descriptor-based likelihood, a candidate with a modest prior but a substantially larger likelihood can accumulate more posterior mass than an incorrectly retrieved top-1 candidate. This Bayesian update enables low-ranked but geometrically consistent poses to overtake retrieval-biased hypotheses; a behavior that correlation-only refinement cannot provide. In high-ambiguity layouts, this principled combination helps improve accuracy without additional geometric solvers or per-scene retraining.

### 3.4. Rotation Invariance via Group Theory

A common error in retrieval-based localization is sensitivity to in-plane camera rotation (roll). Two images of the same location can yield very different descriptors if the representation is not rotation-invariant, which is especially problematic in panoramic or wide FoV settings where roll does not alter the scene layout.

Geometrically, the camera poses are in the Lie group SE(3) of 3D rigid transformations. In-plane rotations form the subgroup SO(2)⊂SE(3), representing rotations about the viewing axis. All poses in this SO(2) subgroup are *orientation equivalent*, as they differ only in viewpoint orientation while preserving the same global layout. SO(2) marginalization is what gives the descriptor its rotation invariance. It removes the effect of in-plane rotation so that identical scenes at different roll angles produce the same descriptor. Since exact marginalization is infeasible, we approximate it efficiently through discrete polar sampling, which is a core component of our method.

Our rotation-invariant circular descriptor is designed to marginalize over this SO(2) subgroup, effectively treating all in-plane rotations of the same scene as equivalent. In mathematical terms, if g(·) denotes the feature extractor applied to an image *I*, we approximate the marginalization

(6) $$\phi \left(I\right)\approx \frac{1}{2\pi }\int_{0}^{2\pi }g\left(R_{\theta }I\right)\;d\theta ,$$

where $R_{\theta }$ represents a rotation of the image by θ degrees. In practice, the feature map is transformed into polar coordinates and sampled using *M* concentric rings with *N* points each. Pooling (average and max) within every ring yields a compact descriptor of size D=M×N×2, and concatenating across rings followed by L2 normalization produces the final rotation-invariant descriptor.

This construction (i) preserves global spatial cues such as the ordering of walls, doors, and structural boundaries while discarding arbitrary in-plane rotations and (ii) enables direct cosine-based similarity between query and rendered views without orientation alignment. By embedding SO(2) invariance into the descriptor, we remove a key nuisance factor from matching, which is especially beneficial in ambiguous layouts where disambiguation relies on large-scale geometry rather than orientation sensitive texture cues. Hence, the system is kept rotation-invariant to in-plane camera spins while still keeping the ability to reason about the larger 3D structure of the scene.

### 3.5. Algorithm Pipeline

- Stage 1: Initialization
  At query time, we begin by semantically segmenting the 3D room mesh and then render a set of panoramic viewports on a uniform 1.2 m × 1.2 m grid, requiring far fewer samples than LaLaLoc’s [23] layout based approach. Inspired by the basal pipeline in [9], each rendered panorama and the input perspective image are passed through pretrained backbones: EfficientNet-S [40] for the query images and DenseNet [41] for the panoramas, to produce dense feature maps.
- Stage 2: Initial Pose Estimation
  We compute depth-wise correlations between the query’s features and each panorama’s features, yielding a similarity score for every candidate viewport. A lightweight MLP takes the top-*k* scoring candidates and directly regresses their 6-DoF poses. All training and evaluation follow the standard ZInD split [12]. This coarse retrieval stage (Figure 2 steps (a) and (b)) efficiently narrows the search from thousands of potential views to a few high-confidence hypotheses and does not require 2D floorplans or low-level annotations as in LASER [10].
- Stage 3: Circular Feature Extraction
  We compute rotation-invariant circular descriptors for the query (and later, for each candidate viewport):
  1. Extract a dense feature map from a pretrained ResNet-50 [11] backbone.
  2. Transform into polar coordinates, sampling *M* rings and *N* points per ring (M=1 and N=8).
  3. Apply average and max pooling per ring to form *D*-dimensional vectors (D=256). Average pooling captures the global structural layout, while max pooling preserves the strong geometric edges. Using them both retains complementary spatial cues essential for disambiguating repetitive scenes.
  4. Concatenate across rings and L2-normalize.
  This stage corresponds to Figure 2 step (c) and produces a compact, rotation-agnostic signature that captures global layout, crucial for disambiguating visually similar scenes.
- Stage 4: Pose Re-ranking
  Finally, as can be seen in Figure 2 step (d), each candidate from Stage 1 is re-rendered and encoded into a circular descriptor. Cosine similarity to the query descriptor is computed, and candidates are re-ranked. Unlike SPVLoc’s correlation-based refinement, which assumes top-1 correctness, our Bayesian combination allows lower-ranked but correct candidates to surface. This avoids failure modes where initial retrieval bias dominates refinement.

## 4. Experimental Evaluation

### 4.1. Baselines

We evaluate Render-Rank-Refine against existing camera pose-estimation baselines (LASER [10] and SPVLoc [9]) on the Zillow Indoor Dataset (ZInD) split [12]. Quantitative performance is measured by translation error (T_err_, cm) and rotation error (R_err_, °) related statistics over all test scenes. Qualitative examples illustrate success cases of our strategy in challenging rooms with overlapping layouts. The GitHub repository for this project is currently under development and will be released in due course. The repositories for SPVLoc and LASER are publicly available online for reference.

### 4.2. Dataset

We validate on the Zillow Indoor Dataset (ZInD) [12], which contains 71,474 panoramas from 1575 unfurnished residential homes, each aligned with its floor plan. ZInD is a well-established, publicly available benchmark that has been widely used in recent research [9,10,23], enabling consistent and comparable performance evaluation. It also encodes features such as open corridors connecting rooms without doors, introducing greater structural diversity in semantic renderings. Its rich 3D annotations allow direct generation of room-level mesh models, which can be converted into semantic meshes either through manual annotation of mesh faces or via learning-based segmentation methods such as PointNet++ [42] or graph neural networks for meshes [43]. Modern frameworks and consumer devices further enable rapid 3D mesh generation without manual annotations or floorplans, such as Open3D [44], LiDAR-equipped smartphones and ARKit-based apps (e.g., Polycam, SiteScape) [45].

### 4.3. Model Training

We initialize all network components with the official SPVLoc [9] pre-trained weights, leveraging their robust semantic and geometric feature representations for faster convergence. The model is fine-tuned once, end-to-end, on our dataset, which is a one-time process that does not require per-scene retraining and is therefore excluded from our computational burden analysis. After a single tuning step on training homes to align the retrieval backbone with general semantics, the model is applied to all test homes without any adaptation, which are all unseen houses with different geometry. To maximize hardware utilization, training uses a batch size of 40 and 24 CPU workers (our system limit). All other training hyperparameters (optimizer, learning rate schedule, etc.) follow the original implementation to ensure consistency and reproducibility. For detailed model hyperparameters and configurations, we direct readers to [9]. Further, minor deviations in error metrics may arise from hardware differences and numerical precision changes (such as float32 instead of float64) necessary for compatibility. We report CPU-only runtime to match SPVLoc [9] and LASER [10], demonstrating lower computational load under identical conditions. With GPU acceleration, our relative advantage would remain because our method relies on lightweight descriptors rather than heavy network inference.

### 4.4. Inference Results

**Quantitative Evaluation** Table 1 presents translation and rotation recall at various thresholds, evaluated on perspective queries with a 90° field of view (FoV). While our recall values closely match those of the refined SPVLoc baseline, minor differences at these coarse thresholds overlook critical variations in *per-case* accuracy. This highlights the need to explore the benefits of rotation-invariant refinement, which is a core step in our pipeline. Rather than focusing on aggregate recall, we break down the maximum, minimum, mean, and median T_err_ (cm) and R_err_ (∘) in Table 2 for a detailed analysis.

Table 2 shows the overall error statistics for all methods. The baseline method has the highest mean T_err_ and R_err_, while refining the baseline reduces both errors. Our method further improves translation accuracy (mean T_err_ = 116.54 cm) while maintaining similar rotational accuracy. Notably, the circular feature-based re-ranking method delivers the lowest mean T_err_ and R_err_ and the smallest median T_err_. Incorporating circular features improves localization accuracy, and further refinement (based on correlation as in [9]) improves performance beyond the baseline and the refined baseline.

**Figure 4:**
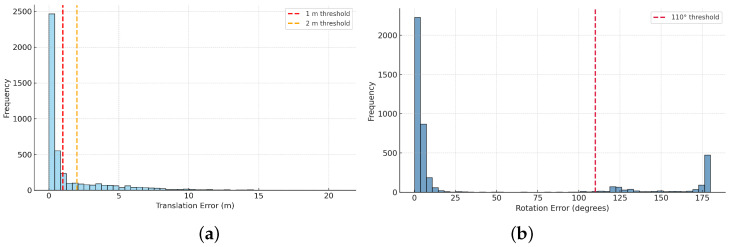
Error distributions on ZInD: (**a**) Translation error distribution with 1 m and 2 m thresholds. Most T_err_ values lie below 1 m with a long tail beyond 2 m. (**b**) Rotation error distribution with a 110° threshold. R_err_ concentrates at small angles, with a secondary mass near extreme flips. Thresholds are chosen from the worst observed T_err_ and a practical failure bound of 110° for rotation.

**Error distribution analysis.** Figure 4 shows the distributions of translation error $T_{\mathrm{err}}$ (m) and rotation error $R_{\mathrm{err}}$ (°) of the baseline on the ZInD test set. To analyze performance, we use thresholds of 1 m and 2 m: the first captures near-accurate cases, while the second brackets the long-tail failures seen in the original pipeline. For rotation, we choose 110° using the same criteria. Most camera poses are predicted within 1 m, but a smaller number produce errors beyond 2 m. Rotation errors are mostly concentrated at small angles, with some outliers corresponding to large orientation flips.

To probe the causes of large errors, we randomly sampled 20 queries beyond each threshold. For $T_{\mathrm{err}}>1$ m, 14 of the 20 (70%) images came from overlapping layouts; for $T_{\mathrm{err}}>2$ m, 13 out of 20 (65%) were overlapping. For severe rotation outliers with $R_{\mathrm{err}}>110{}^{\circ}$, 11/20 (55%) of the sampled images also originated from overlapping layouts. All failure cases point back to structural ambiguity (high A(v)) that can be seen in query images covering more than one room, motivating layout-level disambiguation (e.g., multi-view reasoning or explicit layout priors) in future work. Sampling another 20 queries randomly produced approximately identical results.

**Reverse validation.** To further prove our method’s superior accuracy on query scenarios with high ambiguity, we analyzed the ten scenes with the highest translation errors in the baseline method and examined their corresponding rotation and translation errors. Nearly all of these challenging cases were also found to resemble the ambiguous room layouts as in Figure 1. The mean and median percentage improvements across the hardest cases (top-10 worst $T_{\mathrm{err}}$) are substantial. Compared to the refined baseline, which yields only marginal gains (<1% mean improvement in translation and ∼12% in rotation, with virtually no cases above 90% translation improvement), our method achieves far larger gains: a 40.4% mean and 5.2% median reduction in $T_{\mathrm{err}}$, alongside a 29.7% mean reduction in $R_{\mathrm{err}}$. Notably, 4/10 of these hardest cases reach at least 90% improvement in translation error and 30% achieve this for rotation error, underscoring the robustness of our approach in highly ambiguous settings. On the remaining 6/10 cases, the improvement was found to be more than 50% in both errors. This represents a substantial improvement over the refined baseline (best-case baseline) which achieves such mean gains in rotation error for only 1/10 of the cases and fails to do so in translation accuracy. The top-10 subset analysis is used only to examine extreme ambiguity cases. All quantitative improvements in Table 2 are computed over the full test set. Due to the lack of ambiguity annotations in ZInD, we analyze the worst-case samples as a practical diagnostic; repeating the analysis on the top-20 cases yielded consistent results.

**Figure 5:**
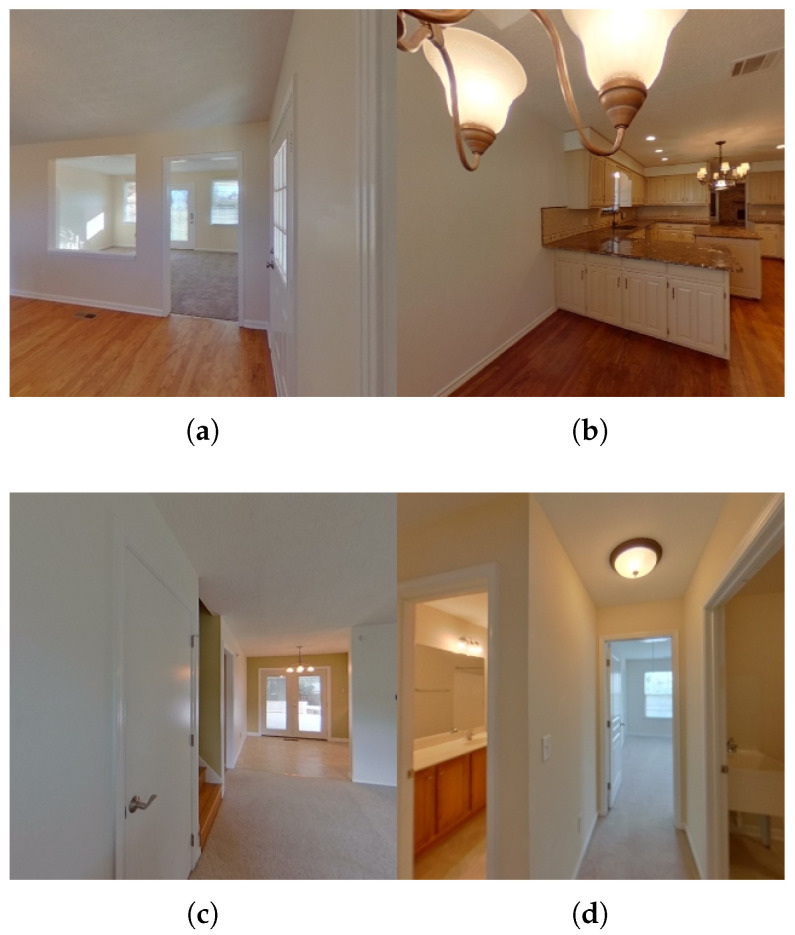
Qualitative results on challenging ZInD [12] scenes. Each illustrates a visually ambiguous layout where baseline methods fail, but our method resolves the camera pose using rotation-invariant circular descriptors. (**a**) Multi-room view. (**b**) Kitchen with occlusion. (**c**) Transitional hallway. (**d**) Hallway between rooms.

**Qualitative Analysis.** In addition to the ambiguous scenes shown in Figure 1, we further examine challenging indoor environments that frequently degrade pose estimation accuracy. Figure 5 shows some representative examples from the ZInD dataset on which our method outperforms the existing state-of-the-art methods of camera pose estimation:

**(a) Multi-room view through cutouts:** Overlapping rooms connected by partial wall openings introduce mixed semantic cues. Our approach successfully isolates the intended viewport by comparing rendered perspectives against the query in a rotation-invariant feature space.

**(b) Kitchen with foreground occlusion:** Complex indoor environments containing strong occlusions (e.g., light fixtures) can obscure discriminative features. The circular descriptors retain robustness to such occlusions, enabling correct disambiguation from visually similar kitchen layouts.

**(c) Transitional hallway in the living room:** Areas connecting multiple rooms present high ambiguity due to overlapping layouts and similar lighting conditions. Our re-ranking step leverages viewpoint-independent features to identify the correct spatial configuration and reject misleading candidates.

**(d) Hallway between a bedroom and living room featuring a bathroom and closet:** The repeated door frames between the rooms and partial bathroom glimpses create visually similar, overlapping layout cues and weak semantics.

**Runtime Analysis.** All experiments were conducted on a workstation equipped with an NVIDIA GeForce RTX 2080 Ti (TU102 Rev. A) GPU with 11 GB VRAM. Nonetheless, all computations used CPU-based multi-threaded processing rather than GPU acceleration. Table 3 compares the runtime characteristics of our method against existing approaches, including PFNet [46] and LASER [10].

Our approach achieves a mean scene sampling time of 0.717 s without refinement and 0.704 s with refinement, representing significant speedup over the baseline and other comparable methods. In terms of inference throughput, our method sustains 25.8–26.8 QPS (queries per second), which is 3 times faster than LASER and more than 5 times faster than PFNet, while maintaining state-of-the-art localization accuracy. QPS is an estimate of how many query images the pipeline can localize per second at runtime, end-to-end. These results show that circular-descriptor re-ranking adds little overhead while sharply reducing sampling time. The use of lightweight features and cosine similarity instead of heavy inference supports near real-time deployment in large indoor environments without loss of accuracy.

## 5. Discussion and Limitation

Our rotation-invariant circular re-ranking improves accuracy and efficiency across settings. Relative to the baseline [9], it cuts mean scene-sampling time by 5.7–5.8 times and sustains 25.8–26.8 QPS, with negligible overhead thanks to lightweight feature extraction and cosine matching (no dense latent rendering). The method also remains reliable in challenging layouts. In ZInD, many queries span overlapping or multi-room views where a single FoV covers distinct spaces that confound viewpoint-specific descriptors. Our rotation-invariant descriptors encode global layout, enabling disambiguation even when local semantics are weak or misleading.

From a Bayesian view, SPVLoc retrieval provides a high-recall but orientation-sensitive prior, while circular descriptors supply a rotation-invariant likelihood; the posterior promotes correct poses without assuming the top-1 candidate, addressing the retrieval-bias failure mode noted in Section 1. Grouping theoretically, treating in-plane rotations as SO(2)⊂SE(3) and marginalizing them via polar sampling yields descriptors that preserve global layout and perform well in symmetric or repetitive scenes. The framework is modality agnostic (such as RGB-LiDAR), requires neither floor plans nor per-scene retraining, and suits latency-sensitive smart health, AR, and robotics.

Our method occasionally underperforms in visually uniform or low-texture scenes (such as plain corridors or doorways with repetitive geometry). In such cases, the rotation-invariant re-ranking may struggle to converge, as the absence of distinctive gradients or depth cues leads to ambiguous feature alignment and local minima. In low-texture regions, circular descriptors rely predominantly on coarse geometric boundaries. Failures primarily occur when heavy occlusions cause ill-defined boundaries in the mesh model rendering at that viewpoint, leading to descriptor insufficiency. Hence, the correct candidate is not found among the top-*k*. Although the pipeline is tolerant to moderate segmentation noise and surface irregularities, since the circular descriptor aggregates global layout, large structural errors (such as missing walls or rooms) can prevent retrieval from obtaining a correct top-k candidate. In cases where vertical and multi-floor ambiguity remains challenging due to limited height cues, incorporating sparse or monocular depth and simple height-based priors (such as wall–ceiling angles) can help.

**Figure 6:**
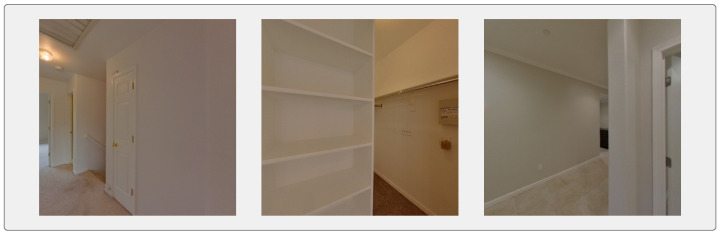
Three representative failure cases illustrating low-texture scenes, repetitive geometry, weak visual cues, and mesh inconsistencies causing render-rank-refine to diverge or worsen the pose estimate relative to the baseline.

Figure 6 illustrates instances where flat, symmetric surfaces reduce descriptor discriminability, degrading pose precision despite an otherwise stable pipeline. Both images fail in our process because the scenes have low texture; the walls, shelves, and doors look plain and have few details. This makes it difficult for the algorithm to extract strong features, so many different camera positions look similar. The scenes also have repetitive geometry, like long walls or similar-looking corners, which creates pose ambiguity; the system cannot tell which position is correct because several poses produce nearly identical images. Refinement then has no clear gradient direction to follow and cannot improve the estimate. These areas also have mesh errors, such as missing depth on narrow walls or shelves, which causes the rendered view to not match the query image and further weakens the refinement step. Vertical or multi-floor ambiguity also persists due to the absence of reliable height cues.

## 6. Conclusions

We present Render-Rank-Refine, a two-stage framework for robust indoor camera pose estimation. The approach treats candidate retrieval as a high-recall prior and circular descriptor matching as an ambiguity-resolving likelihood, making the final refinement a coherent probabilistic update. From a group-theoretic perspective, marginalizing in-plane rotations removes a nuisance variable while preserving the global spatial layout, thereby reducing retrieval bias and stabilizing pose estimates in repetitive environments. On the Zillow Indoor Dataset, our method achieves substantial speedups over prior approaches while maintaining or improving accuracy, with the most significant gains observed in ambiguous layouts. The framework is efficient, lightweight, and modality-agnostic, naturally extending beyond RGB to cross-domain scenarios, making it well suited for real-time applications in AR, robotics, and assistive systems. Our method is generally robust but may struggle in low-texture or heavily occluded scenes, and major errors from mesh models can prevent correct retrieval. Future work will focus on improving robustness to illumination, occlusion, and vertical ambiguity, with potential gains from adding depth cues and simple height-based priors. We also plan to evaluate on self-collected perspective datasets along with app-generated semantic meshes, and extend experiments beyond residential settings to commercial and healthcare environments for broader generalization.

## Figures and Tables

**Table 1:** Translation and rotational recall (%) at varying thresholds for different pipeline variants. SPVLoc-Refined and Ours-Refined refer to the baseline with one step of correlation-score-based refinement from [9].

Method	<10 cm (%)	<50 cm (%)	<1 m (%)
LASER (from [9])	8.69	67.01	80.90
SPVLoc	12.25	59.49	98.21
SPVLoc–Refined	22.07	73.80	98.21
Ours	12.86	63.42	99.14
Ours–Refined	21.82	73.62	99.14

**Table 2:** Overall translation (cm) and rotation (°) error statistics for all approaches.

Method	*T*_err_ (cm)				*R*_err_ (°)			
	Min	Max	Mean	Median	Min	Max	Mean	Median
Baseline	0.406	2089.24	149.93	32.38	0.040	179.99	38.91	3.57
Baseline–refined	1.210	2069.46	131.73	18.47	0.046	179.99	36.33	2.97
Ours	0.406	2089.24	116.54	29.17	0.040	179.99	35.98	3.44
Ours–refined	0.406	2069.47	103.07	19.41	0.027	179.99	30.20	2.56

**Table 3:** Runtime comparison of scene sampling time (mean ± std, seconds) and inference throughput (queries per second, QPS). Bold text indicates best performance.

Method	Scene Sampling Time (s)	Inference QPS
PFNet [46]	48.95 ± 38.95	5.06
LASER [10]	0.97 ± 1.09	8.31
Baseline [9]	1.66 ± 1.06	**28.63**
Ours	**0.72 ± 0.65**	25.79
Ours (refined)	**0.70 ± 0.63**	26.39

## Data Availability

The data presented in this study are openly available in ZInD (https://github.com/zillow/zind (accessed on 12 November 2025)) at DOI:10.1109/CVPR46437.2021.00217.
